# Nanoindentation Induced Deformation and Pop-in Events in a Silicon Crystal: Molecular Dynamics Simulation and Experiment

**DOI:** 10.1038/s41598-017-11130-2

**Published:** 2017-08-31

**Authors:** Sun Jiapeng, Li Cheng, Jing Han, Aibin Ma, Liang Fang

**Affiliations:** 1 College of Mechanics and Materials, Hohai University, Nanjing, 210098 Jiangsu Province PR China; 2 School of Mechanical and Electrical Engineering, China University of Mining and Technology, Xuzhou, 221116 Jiangsu Province PR China; 3 State Key Laboratory for Mechanical Behavior of Materials, Xi’an Jiaotong University, Xi’an, 710049 Shaanxi Province PR China

## Abstract

Silicon has such versatile characteristics that the mechanical behavior and deformation mechanism under contact load are still unclear and hence are interesting and challenging issues. Based on combined study using molecular dynamics simulations and experiments of nanoindentation on Si(100), the versatile deformation modes, including high pressure phase transformation (HPPT), dislocation, median crack and surface crack, were found, and occurrence of multiple pop-in events in the load-indentation strain curves was reported. HPPTs are regard as the dominant deformation mode and even becomes the single deformation mode at a small indentation strain (0.107 in simulations), suggesting the presence of a defect-free region. Moreover, the one-to-one relationship between the pop-in events and the deformation modes is established. Three distinct mechanisms are identified to be responsible for the occurrence of multiple pop-in events in sequence. In the first mechanism, HPPTs from Si-I to Si-II and Si-I to bct5 induce the first pop-in event. The formation and extrusion of α-Si outside the indentation cavity are responsible for the subsequent pop-in event. And the major cracks on the surface induces the pop-in event at extreme high load. The observed dislocation burst and median crack beneath the transformation region produce no detectable pop-in events.

## Introduction

Single crystal silicon is the most important material in semiconductor industry and in micro/nanoelectromechanical systems (MEMS/NEMS). Exploring and understanding the mechanical behavior and deformation mechanism are critical to the design, production and operation of Si-based structures and devices. In the past half-century, micro/nano-indentation has been extensively applied to investigate this matter both experimentally and theoretically. In many of those studies, silicon, which is a completely brittle material at room temperature, displays a distinct ductile behavior resulting from the high-pressure phase transformation (HPPT)^1–4^, slip^1, 5^, planar defect^6^, twinning^7, 8^ or dislocations^3, 9, 10^ under a contact load. Due to the complex nature of silicon, complete understanding of its plastic deformation is still a challenging issue and hence an interesting research topic.

Early studies have shown that HPPT is the dominant mode of plastic deformation in single crystal silicon, followed by brittle fracture above a certain load under micro-indentation up to a large temperature (~370 °C~500 °C)^11–13^. Beyond that temperature range, the plastic deformation is dominated by the dislocation activity. Subsequently, the HPPT of single crystal silicon under micro/nano-indentation are extensively studied.

*In situ* electrical measurements show a transformation from a semiconductor-like phase (pristine diamond cubic structure, Si-I) to a metallic phase^14, 15^. The resulted metallic phase is generally accepted to be the β-Si phase, which is a body-centered-tetragonal structure with sixfold coordination, based on the phase transformation sequences observed in diamond-anvil cell (DAC) experiments^15^. This transformation may lead to an abrupt change in the load-displacement curve, which is called a ‘pop-in’ event. During unloading, sudden changes in both the contact electrical resistance and the load-displacement curve (known as a ‘pop-out’) indicate further phase transformation, which is believed to be the transformation from the Si-II phase to the crystalline bc8/r8 phase^16, 17^. A slow change in the load-displacement curve (known as an elbow) indicates the formation of amorphous silicon (α-Si). Further investigations have demonstrated that the HPPT and resultant mechanical behavior during micro/nanoindentation are closely related to the maximum indentation load^17–19^, load/unload speed^16–20^, geometry of the indenter^17, 21^ and the crystallographic orientation of the indented surface^4, 22, 23^.

Recently, Wong *et al*.^2^ reported that the HPPT could be the single deformation mechanism without extensive damage under a relatively small maximum load using a sphere indenter, but no pop-in event was found. Upon increasing the maximum load, extensive damage (major slip, cracking and crystalline damage) is observed under the indenter. The authors also investigated the mechanism of initial plastic deformation with respect to the hold time under the maximum load, and suggested that plastic deformation is typically initiated by HPPT or crystalline defect^24^. These results indicate that defect propagation and HPPT are mutually exclusive, competing deformation mechanism.

Most researchers have reported that crystalline defects, including slip bands, planar defects and dislocations, and cracks commonly coexist with HPPT under micro/nanoindentation of single crystal silicon, as determined by transmission electron microscope (TEM)^3, 5–7, 16, 21, 25, 26^. The common feature of the cross-sectional TEM (XTEM) images of deformation region is that phase transformation region locates on the topmost of the indented surface, followed by the crystalline defects and cracks underneath the transformation region^1, 2, 16, 24^. The deformation mechanism of single crystal silicon is closely related to load conditions. Under some load conditions, such as the compression of single crystal silicon nanoparticle^10, 27^ or submicron pillar^28, 29^, dislocations were observed to be the single form of plastic deformation. On scratching and grinding process, the α-Si on the topmost of the scratched surface and the damaged crystalline Si with high densities of dislocations underneath the α-Si feature the deformation region^30–33^, and an alternative phase transformation route is suggested: Si-I → α-Si → Si-III/Si-XII in α-Si, which is quite different from that in the case of nanoindentation (i.e., Si-I → Si-II → Si-III/Si-XII or α-Si). Recently, nanoscale solely amorphous layer, followed by pristine crystalline lattice, is obtained under ultraprecision grinding using the newly developed diamond wheel with ceria^34^.

Molecular dynamics (MD) simulations are also widely used to study the mechanical behavior^35–38^ and HPPT^22, 36, 39–43^ of single crystal silicon during nanoindentation. The MD simulations successfully predict the formation of the Si-II phase and show the detailed distribution, structural characteristics and phase transformation process of Si-II^36, 39–46^, which is almost impossible to achieve in present experimental conditions. In addition, another new body-centered-tetragonal high-pressure phase of bct5 with fivefold coordination is predicted by the MD simulations^41, 42, 45^. Recently, an *in situ* Raman micro-spectroscopy experiment confirmed the existence of the bct5 phase during nanoindentation^47^. Thus, MD simulations have become an indispensable approach to furthering our understanding of these processes at the atomic scale.

In this paper, we performed large-scale MD simulations and experiment study of nanoindentations on (100)-oriented silicon [Si(100)]. We focused on the indentation-strain-dependent mechanical behavior and complicated deformation mediated by HPPT, dislocations and brittle cracking. The versatile deformation mechanisms of single crystal silicon were investigated, and occurrence of multiple pop-in events in the load-indentation strain curves was found. Moreover, three distinct mechanisms were identified to be responsible for the successive occurrence of pop-in events. The process of HPPT, the distribution of high-pressure phases, the nucleation and propagation of dislocations, and brittle cracking were further investigated in details. This work provides new insight into the mechanical behavior and versatile deformation mechanisms of single crystal silicon, which advances our understanding of the nature of silicon material.

## Methods

### MD simulations procedure

**Figure 1:**
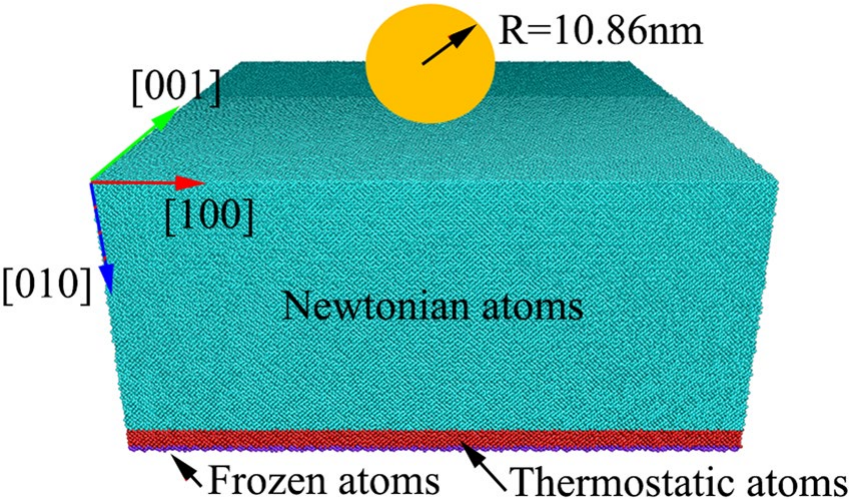
Molecular dynamics simulation model.

Large-scale MD simulations were performed to investigate nanoindentation on a flat Si(100) surface. A newly developed screened empirical bond-order potential (a screened version of the potential developed by Erhart *et al*.^48^) was used^49^, as this potential provides the improved descriptions of the mechanical behavior of nanowires under uniaxial tension and the deformation mechanisms during cutting of monocrystalline and polycrystalline silicon^50^. Figure 1 shows the adopted MD simulation model. In this model, a virtual spherical indenter with a diameter of ~21.72 nm was use, which was modeled using the repulsive force: *F* = *AH*(*r*)(*R-r*)^2^, where *A* is the force constant with value of 10 eV/A^2^, *H*(*r*) is a step function, *R* is the indenter radius, and *r* is the distance from the silicon atoms to the center of the indenter sphere. The single crystal silicon specimen had dimensions of 43.45 × 43.45 × 29.87 nm^3^ and contained approximately four million atoms. To obtain the equilibrium configuration of the silicon specimens at a temperature of 300 K, a meticulous heat treatment process was performed, in which the temperature was controlled using the NVT ensemble with Langevin dynamics^51^. The initial specimens, composed of silicon atoms in a diamond cubic structure with a lattice parameter of 5.431 Å, were first heated from 0 K to 600 K over 25 ps and then at this temperature for 25 ps. Then, the heated specimens were gradually cooled to 300 K over 25 ps, subsequently followed by annealing at 300 K over 50 ps. Elaborately heating the sample to an elevated temperature of 600 K was performed to speed the equilibration process.

After the initial construction, MD simulations of nanoindentation were performed at a constant velocity of 80 m/s by the LAMMPS code^52^. The free boundary was applied along the indentation direction, while periodic boundary conditions were applied along the other two directions. The atoms in a layer with a thickness of 0.5 nm was frozen to provide structural stability on the bottom of the specimen. The next atom layer with a 2 nm thickness adjacent to frozen layer was maintained at a constant temperature of 300 K to dissipate excess thermal energy. All the remaining atoms were allowed free movement according the Newtonian motion equations.

The combination of the modified coordination number (MCN), considering the first and second nearest neighbors, the radial distribution function (RDF); and the bond angle distribution function (ADF) was applied to identify five crystal phases (Si-I, Si-II, Si-III, Si-XII, and bct5) and amorphous phase (α-Si) observed in the previous studies. The structure characteristics of five crystal phases are summarized in Table S1 in supplementary material along with the typical transition pressure. According to the authors’ previous work, the isolated Si-III/Si-XII atoms around the transformation region were identified as a slightly distorted diamond cubic structure (DDS) rather than the Si-III/Si-XII phase structure. A detailed description of the combined approach can be found in the supplementary material and in our prior work^45, 53^.

## Experimental

### procedure

A single crystal silicon (100) sample was prepared from an intrinsic silicon wafer (320 μm thickness). The root-mean-square (RMS) roughness of the sample was measured as around 0.5 nm. Before tests, the sample was dipped into 5 wt.% hydrofluoric acid for 2 min to remove the superficial native oxide layer. Then, the sample was ultrasonically cleaned with acetone, ethanol and deionized water in sequence for 5 minutes to remove surface contamination. Many researchers showed that this procedure is able to prepare a contamination free bare silicon surface^54, 55^.

The nanoindentation tests were performed using a NanoTest Vantage (Micro Materials Ltd.) with a diamond spherical indenter of 1 μm radius. The sample was subjected to the peak load of 3 mN, 10 mN, and 15 mN at a fixed loading rate of 0.1 mN/s, held at peak load for 20 s to minimize the time-dependent plastic effect, then unloaded at 0.1 mN/s. For each condition, 8 tests were repeated. After tests, a field emission scanning electron microscope (SEM, Zeiss) was used to examine the morphology of residual impression.

## Simulation Results

### Mechanical behavior

**Figure 2:**
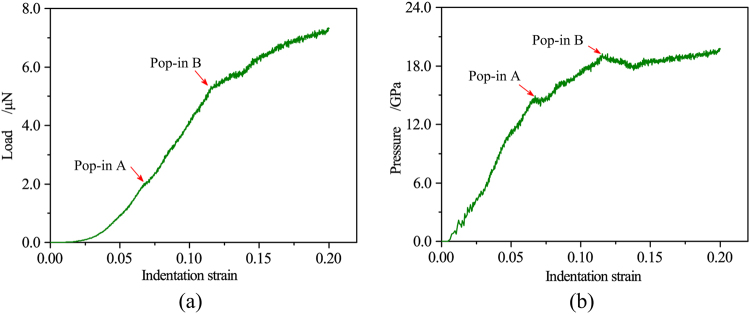
(**a**) *L-ε* curve and (**b**) *P*_*m*_ *-ε* curve during nanoindentation on Si(100) predicted by the MD simulation. The red arrows mark the pop-in events.

The characteristic indentation strain, $\,\varepsilon =0.2(h/a)$ was took the place of the generally used indentation depth, where *h* is the contact depth and *a* is the contact radius. The resultant load-indentation strain (*L-ε*) curve (Fig. 2(a)) illustrates the representative elastic-plastic response of single crystal silicon to nanoindentation. A key interesting result is that two distinct pop-in events are detected in the *L-ε* curve at an indentation strain of *ε*_*A*_ = 0.067 and *ε*_*B*_ = 0.115. Although the occurrence of multiple pop-in events has been reported in some experimental studies^2, 18, 22^, this is the first report, to the best of our knowledge, of multiple pop-ins predicted by MD simulations.

The mean contact pressure was also calculated with respect to the indentation strain, as shown in Fig. 2(b), which is defined as *P*_*m*_ = *P/πa* ^2^, where *P*_*m*_ is the mean contact pressure, *P* is the load, and *a* is the radius of the circle of contact. The *P*_*m*_ *-ε* curve provides the characteristic values of 14.91 GPa and 19.22 GPa, which mark the onset of pop-in A and pop-in B, respectively. The Pop-in A is generally found in experiments and is widely accepted to indicate the initial HPPT from Si-I to Si-II^2, 5, 18, 24, 51^, which suggests the onset of plastic deformation. The predicted pressure for Pop-in A is consistent with the experimental result of 9.9–15GPa^22, 56^ and the theoretical value for HPPT from Si-I to Si-II^57^.

### High-pressure phase transformation

**Figure 3:**
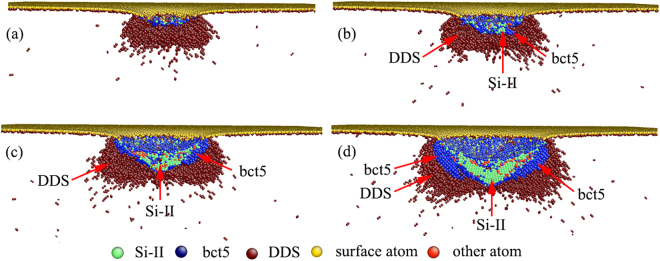
Side cross-sectional views of the phase distribution at *ε* = (**a**) 0.060, (**b**) 0.067, (**c**) 0.080, and (**d**) 0.095.

The sequence of snapshots in Fig. 3 illustrates the evolution of high-pressure phases at indentation strains of 0.060, 0.067, 0.080 and 0.095, showing the cause of Pop-in A. In Fig. 3(a), although some Si-II and bct5 atoms are observed at *ε* = 0.060, these atoms are only the isolated atoms and not a cluster of a single phase. The first detectable HPPT from Si-I to Si-II occurs at *ε* = 0.067 [Fig. 3(b)] when the mean contact pressure reaches its local maximum of 14.91 GPa, and Pop-in A occurs. Almost at the same time, a bct5 phase forms around the Si-II phase. As the penetration proceeds, the Si-II phase continually expands to a large single-phase volume beneath the indenter, and the bct5 phase extends around the Si-II phase region, as illustrated in Fig. 3(c) and (d). The entire transformation zone is immersed in the DDS region, which indicates the existence of a distorted lattice in this region.

The Si-I to Si-II transformation leads to a 23% decrease in volume, while only a 15% decrease in volume occurs during the Si-I to bct5 transformation. The sudden volume reduction of the constrained region beneath the indenter results in a sharp load reduction of the indenter, *i.e*., a pop-in event, when the Si-I to Si-II and Si-I to bct5 transformations occur. Moreover, the Si-I to Si-II transformation is caused by flattening the tetrahedral structure along the [001] direction, and the Si-I to bct5 transformation is formed by flattening the stepped sixfold rings of the diamond lattice^41^. Hence, although both transformations from Si-I to bct5 and from Si-I to Si-II, can induce a pop-in, we suggest that the Si-I to Si-II transformation is the primary contributor to Pop-in A.

**Figure 4:**
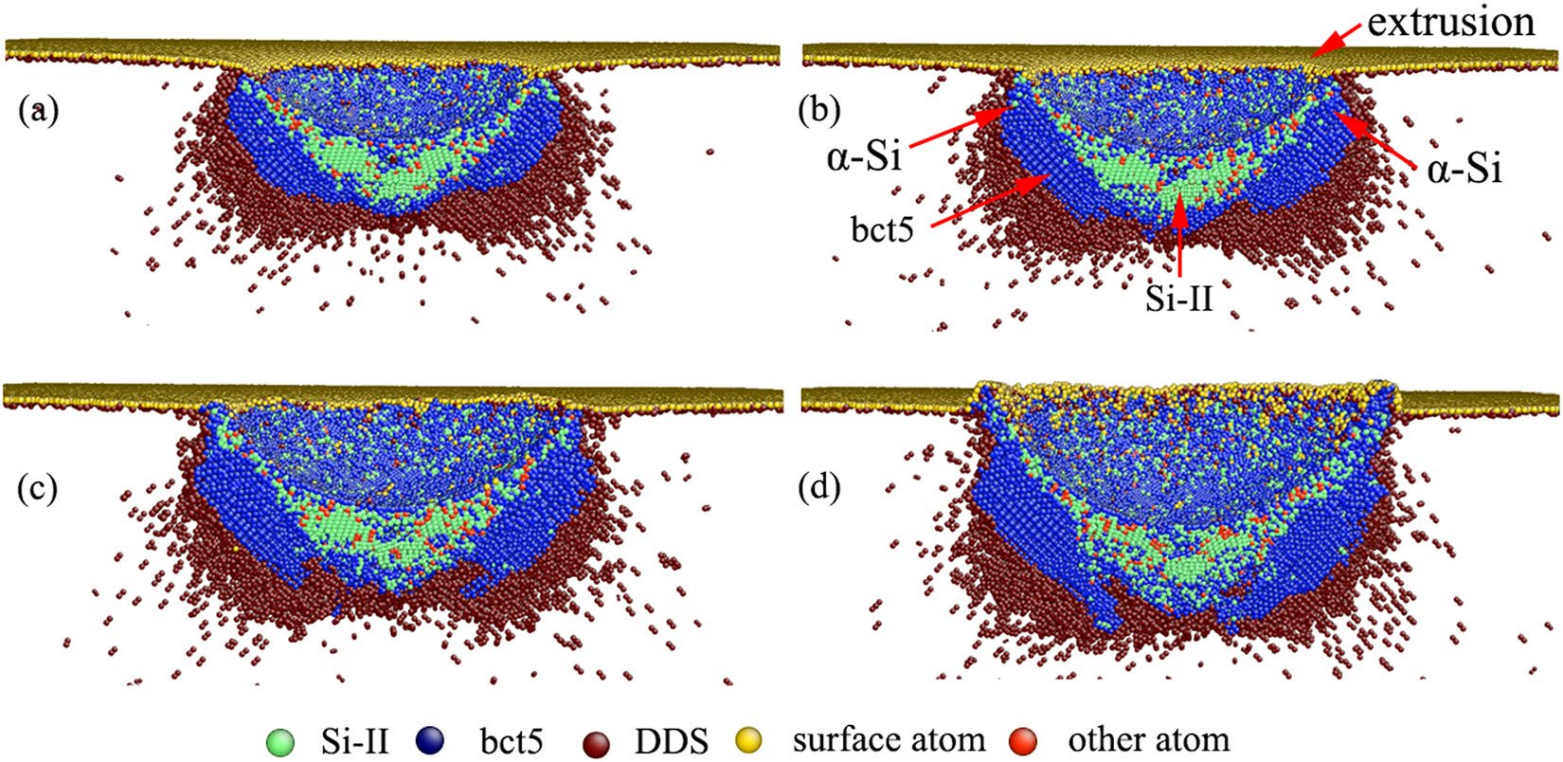
Side cross-sectional views of the phase distribution at *ε* = (**a**) 0.109, (**b**) 0.115, (**c**) 0.124, and (**d**) 0.138.

**Figure 5:**
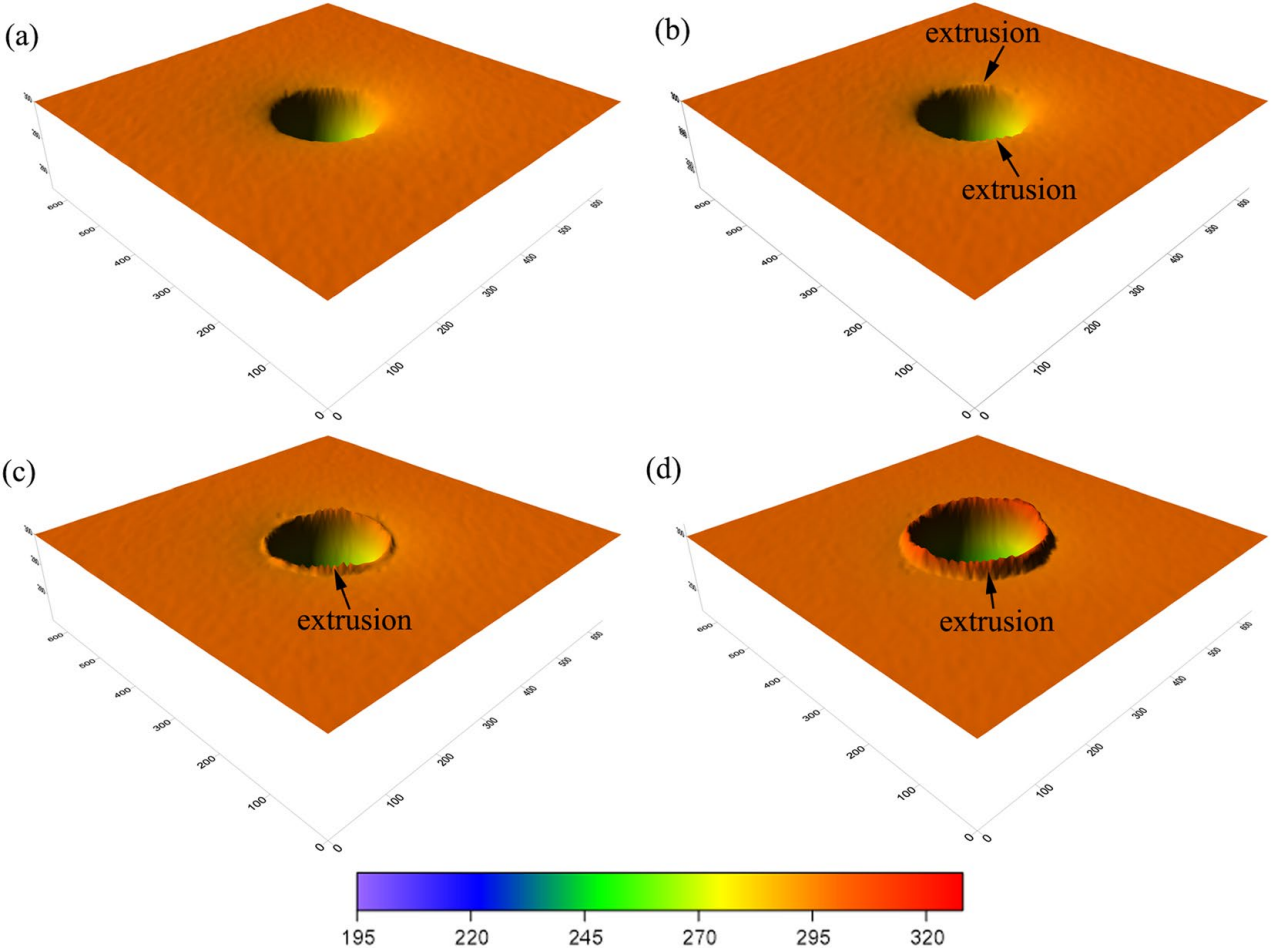
The surface morphology of impression at *ε* = (**a**) 0.109, (**b**) 0.115, (**c**) 0.124, and (**d**) 0.138.

As the nanoindentation proceeds, pop-in B occurs at the indentation strain of 0.115, as shown in Fig. 2. Note that pop-in B is a kink pop-in and is significantly different from pop-in A. For pop-in A, the load sharply drops. In contrast, a gradual load increase and pressure reduction are found for pop-in B, which is similar to the kink pop-out generally found in experimental nanoindentation during unloading^22^. Figure 4 illustrates the structure evolution at different indentation strains of 0.109, 0.115, 0.124 and 0.138. We note that the single crystal Si-II and bct5 phases are the only two high-pressure phases when the indentation strain is less than 0.115, as shown in Figs 3 and 4(a). When the indentation strain increases over the critical value of 0.115, the amorphous silicon (α-Si), which is the mixture of atoms with coordination number equal to 4, 5 and 6, appears around the indenter. Simultaneously, the continuous extrusion of α-Si outside the indentation cavity is detected, as illustrated in Figure 4(a), indicating that the transformed region extends beyond the constraint of the indenter. The transportation of α-Si to the surface causes the gradual pressure reduction reflected by the kink pop-in event. This result demonstrates that the formation and extrusion of α-Si outside the indentation cavity is responsible for pop-in B in the *L-ε* curve. Further evidence can be obtained from the 3D surface morphology of impression, as shown in Fig. 5, which shows an unequivocal link between α-Si extrusion and pop-in B. Interestingly, the occurrence of pop-in B is resulted from the pressure-induced amorphization and is facilitated by the extrusion of α-Si. Therefore, pressure-induced amorphization without extrusion can also promote the occurrence of pop-in, which was confirmed by our previous MD simulations using the Tersoff potential^58^.

**Figure 6:**
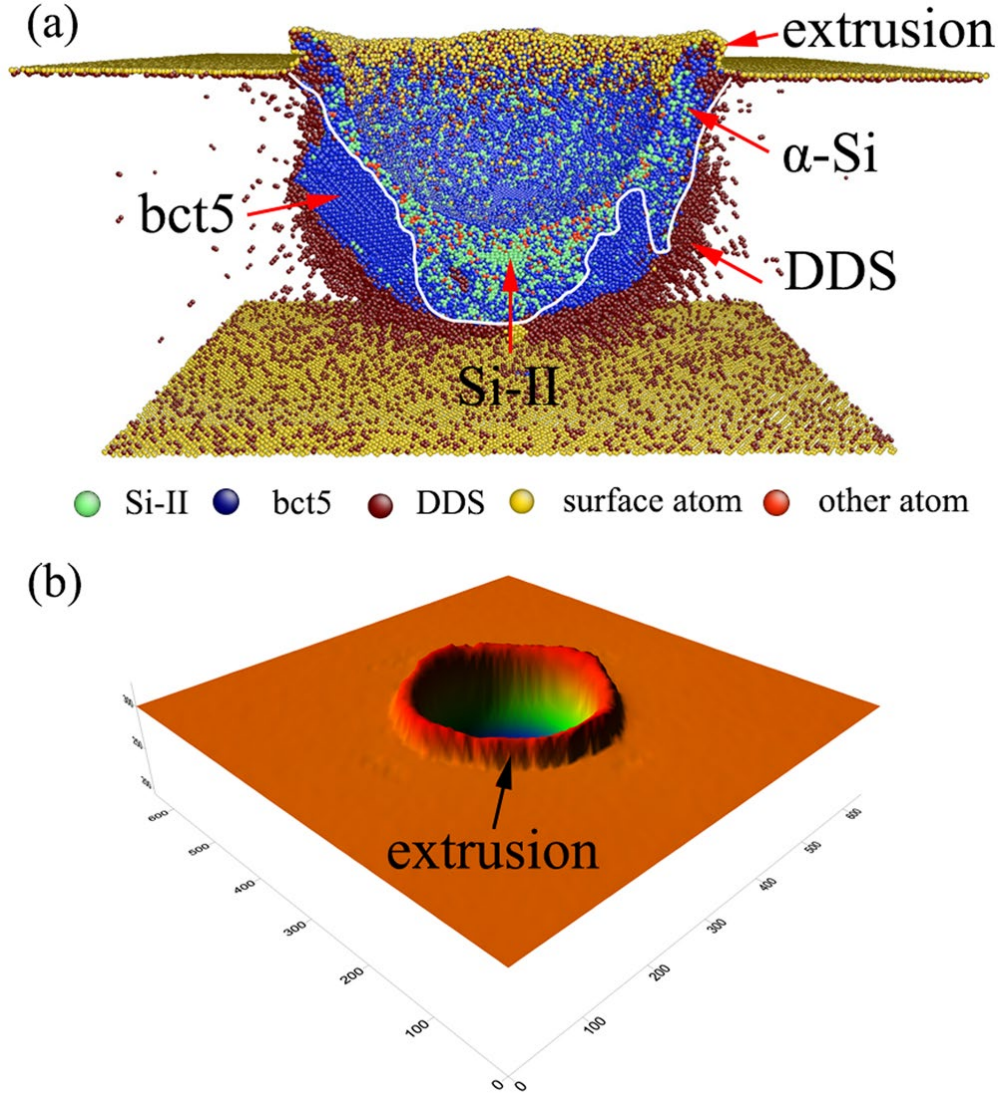
The high-pressure phases distribution and the surface morphology at *ε* = 0.2.

As the nanoindentation strain continues to increase, the Si-II and bct5 phases gradually transform into α-Si, and the α-Si continuously extrudes outside the indentation cavity [Figs 4(c,d and 5c,d)]. In particular, the single crystal Si-II nanovolume beneath the indenter is also gradually replaced by α-Si [Fig. 4(d)]. Therefore, amorphous silicon becomes the dominant high-pressure phase when the indentation strain approaches an extreme large value, as illustrated in Fig. 6. In this Figure, a large amorphous zone distributes directly underneath the indenter, and a large α-Si extrusion forms on the surface. The bct5 phase is located around the amorphous silicon. Almost all of the Si-II formed at small *ε* transforms into amorphous silicon. Moreover, the bct5 phase is abundant at any indentation strain and is comparable with Si-II at small *ε* and with α-Si at large *ε*, which suggests more attentions should be given to the bct5 phase. Consequently, the complete scenario of high-pressure phases evolution during nanoindentation on Si(100) is elucidated.

More detailed structure characterizations of Si-II, bct5 and α-Si were obtained by calculating the RDF and ADF within the congregating single-phase region identified by MCN, as shown in Figures S1, S2, S3, and S4 in supplementary material.

### Dislocations during the nanoindentation process

**Figure 7:**
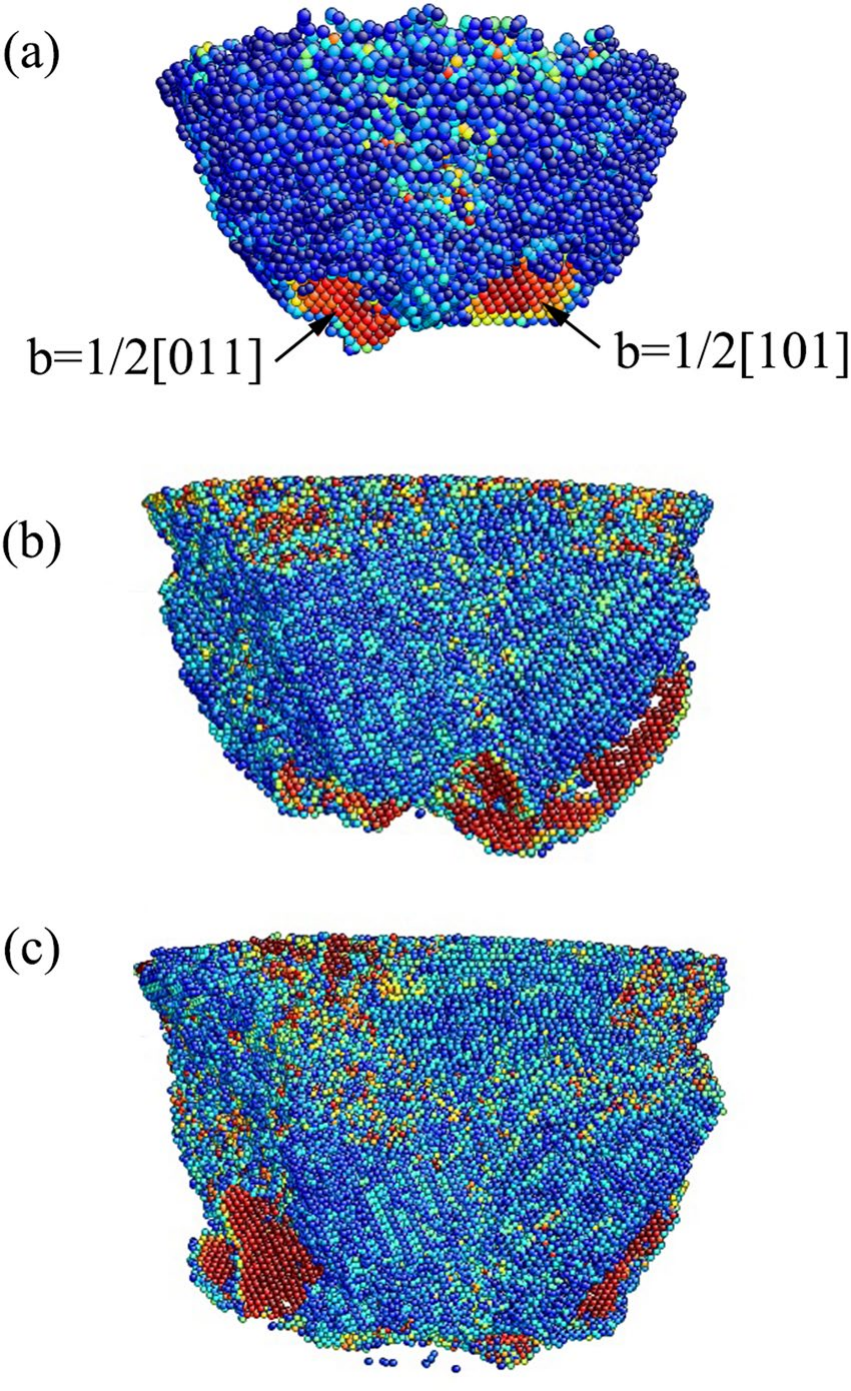
Slip vector analysis of the dislocation structure at *ε* = (**a**) 0.107, (**b**) 0.138, and (**c**) 0.2.

Two full dislocations with Burger vectors 1/2[011] and 1/2[101] nucleate beneath the phase transformation region at *ε* = 0.107, as shown in Fig. 7(a). With an increase in the indentation strain, these dislocations move in its slip plane toward the untransformed region, and new full dislocations nucleate and following glide continually [Fig. 7(b,c)]. All the dislocations are blocked by the phase interface. That is, the dislocations glide in the untransformed region around the transformation region. This becomes in accordance with the experimental result in the literature^1–3, 24, 59^. Generally, dislocation burst can lead to sudden, repeated unloading and loading cycles, leaving many serrations in the *L-ε* curve for metal^60^. However, here, this dislocation behavior does not leave any trace in the *L-ε* curve. One reason is that the resultant dislocations are located far away from the indenter. The other reason lies in the lack of sufficient dislocations. In fact, even at the maximum indentation strain, we observe only a few of dislocations, as shown in Fig. 7(c). Consequently, the dislocations only slightly contribute to the plastic deformation in the present condition.

### Cracking during the nanoindentation process

**Figure 8:**
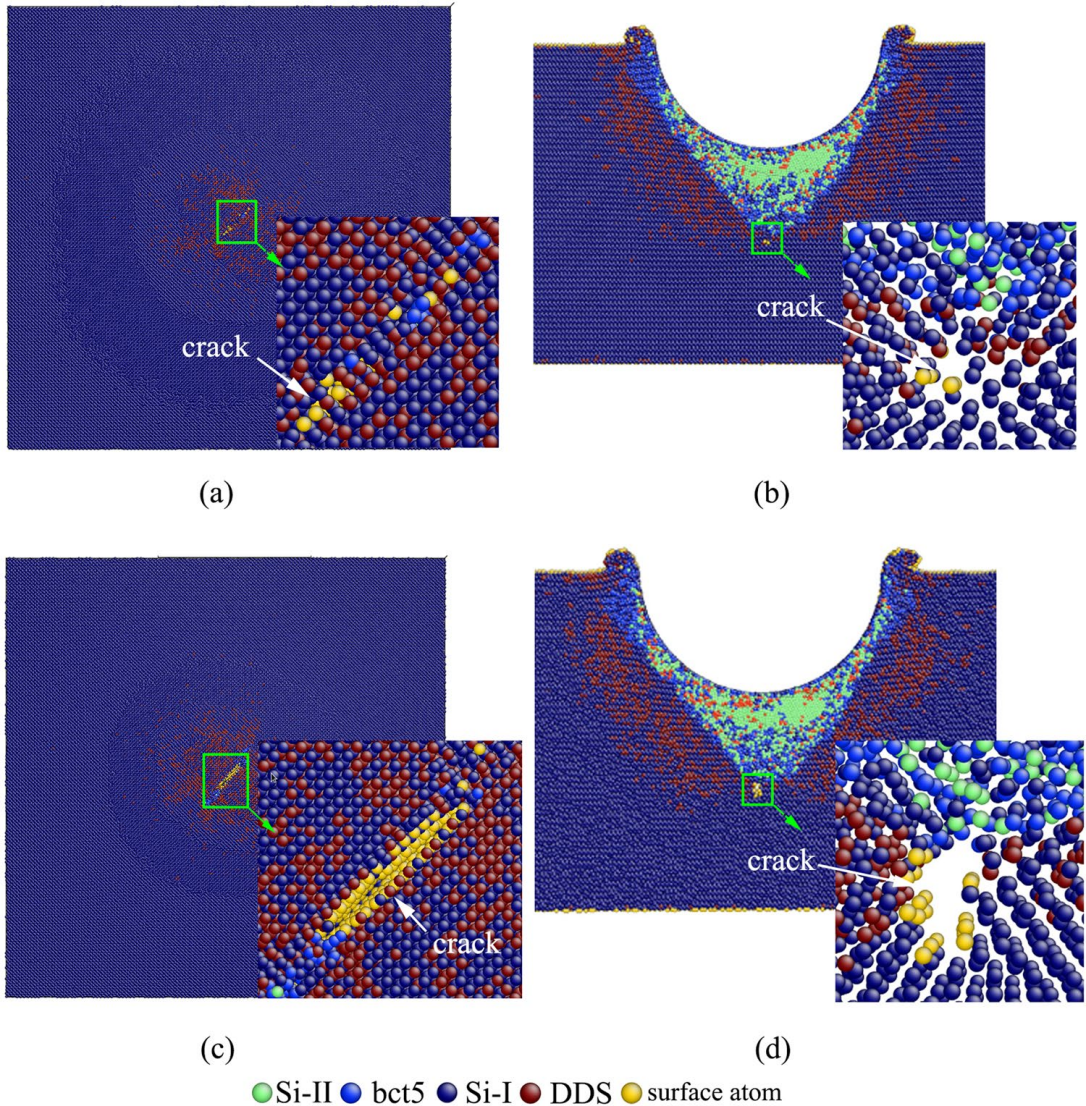
Cross-sectional view (**a**), (**c**) and side cross-sectional view (**b**) and (**d**) of crack evolution at *ε* = (**a**), (**b**) 0.185, and (**c**), (**d**) 0.2.

A median crack along the [110] orientation is emanating from the bottom of the phase transformation region, as shown in Fig. 8(a) and (b), when *ε* increases to above 0.185. The crack nucleates in the irregular α-Si/Si-I phase interface at the bottom of the transformation region, as illustrated in Fig. 8(a). The median crack is generally found during nanoindentation on Si(100) with a spherical indenter when the indentation load increases^25^, and becomes even more widespread using Berkovich indenter^21^. Although the median crack was reported to nucleate at the defects intersection, no link between the crack nucleation and the defects intersection is found. With a continuous increasing in *ε*, the median crack extends down into the untransformed region, as illustrated in Fig. 8(c) and (d).

This result indicates silicon tends to brittle deformation at large indentation strain during nanoindentation. To the best of our knowledge, this is the first successful prediction of median crack during nanoindentation on silicon by the MD simulations, although cracking has been proven to occur based on experiments at a large indentation strain, as indicated in our subsequent experiment and reported in the literature^21, 25, 59^. The crack-driven brittle deformation is also observed during the cutting of polycrystalline silicon using MD simulation^50^.

## Experimental Results

**Figure 9:**
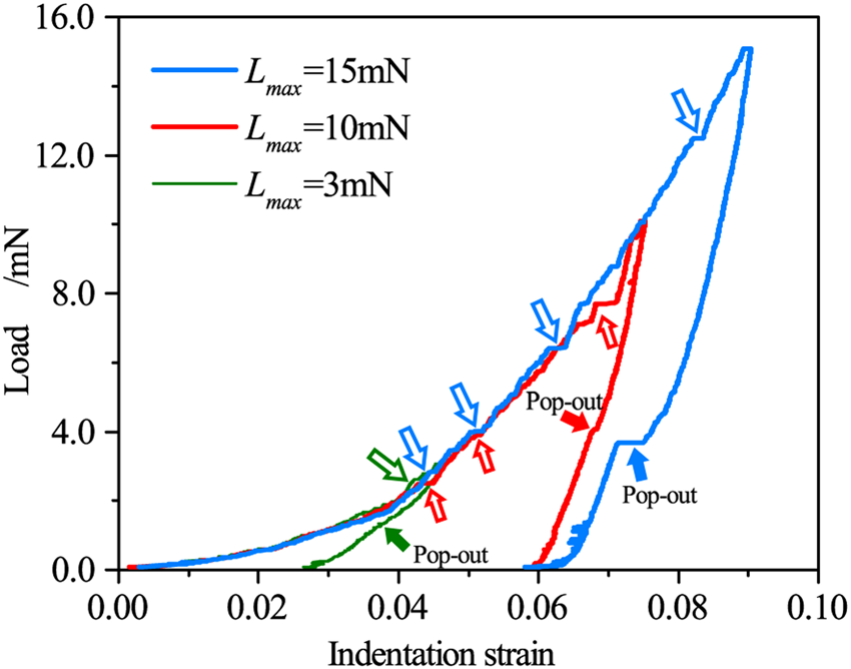
Experimental *L-ε* curve during nanoindentation on Si(100). The open arrow marks the pop-in events and solid arrow marks the pop-out events.

Figure 9 shows the typical experimental *L-ε* curves of Si(100) during nanoindentation at different peak load. The occurrence of multiple pop-in events clearly appears in the *L-ε* curve during loading, providing convincing evidence for the present MD simulations. The number of pop-in events increases with an increase in peak load. At small peak load of 3 mN, only one pop-in event is found at *ε* = 0.042, indicating onset of non-elastic deformation at low load. At large peak load of 10 mN and 15 mN, three and four pop-in events are detected in the *L-ε* curve. The initial pop-in, indicating the onset of HPPT and transition from purely elastic to elastic-plastic deformation, occurs over a small strain range of 0.042~0.044 for all three peak loads. The initial pop-in pressure, *i.e*. HPPT pressure, was calculated over a small range of 9.24~10.06 GPa, which is consistent with the experimental result of 9.9–15GPa^22, 56^ reported in the literature. This initial pop-in strain and pressure is smaller than values of 0.067 and 14.91 GPa predicted by the present MD simulation. This discrepancy between the experiment and MD simulation due to the large difference in indenter size and indentation velocity. The increased pop-in load with loading rate has been reported for the single crystal silicon^61^.

The second pop-in event at peak load of 10 mN and 15 mN appears at nearly same indentation strain of 0.062. The different strain for the third pop-in event at peak load of 10 mN and 15 mN is observed, indicating experimental scatter as shown in Fig. 9. At large peak load of 15 mN, the fourth event is observed at a large indentation strain of 0.082. During unloading, the pop-out and elbow appear statistically for all the tests, showing further phase transformation. The pop-out events and its mechanism have received much research attention, but are beyond the scope of this article.

**Figure 10:**
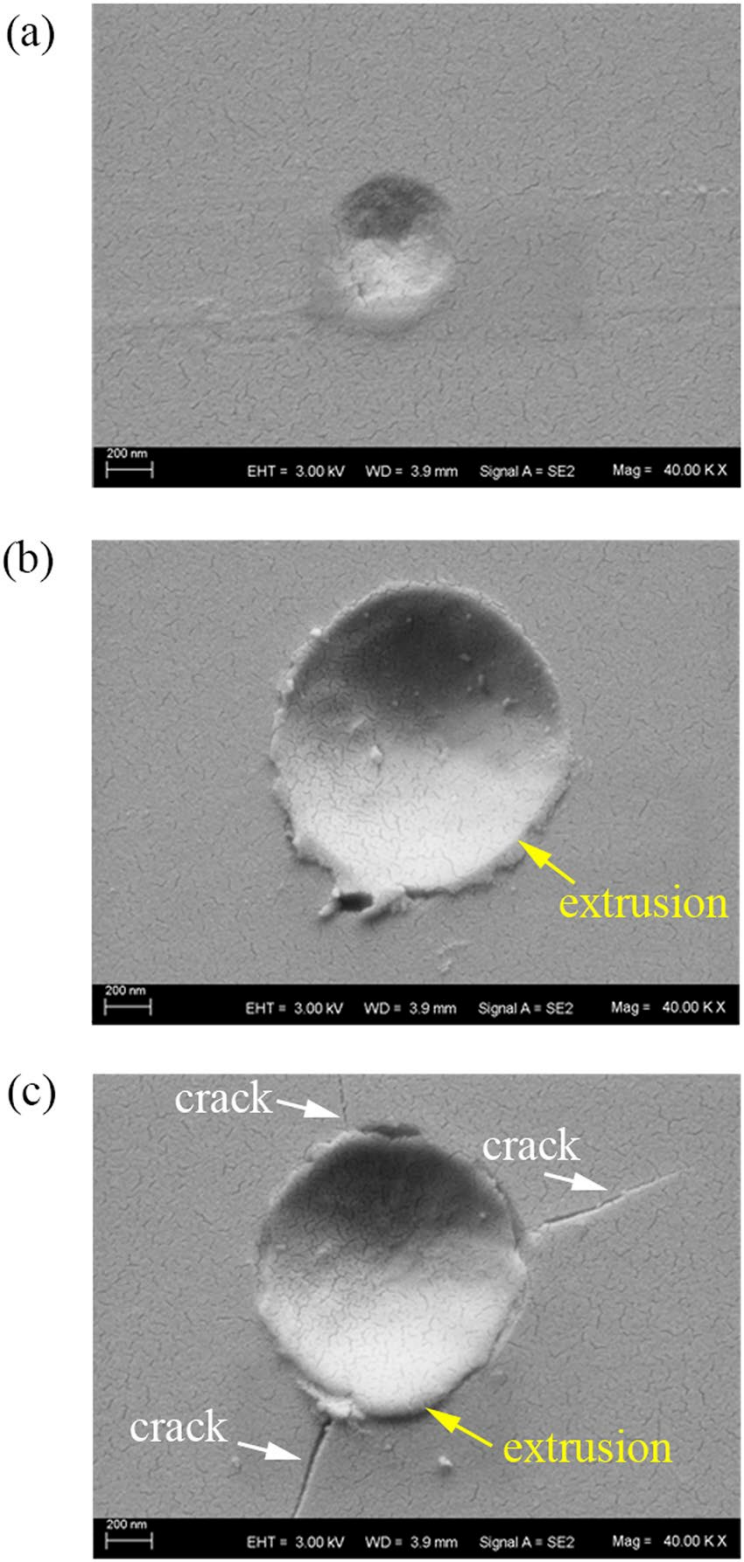
The morphology of residual impression at peak load of (**a**) 3 mN, (**b**) 10 mN, and (**c**) 15 mN.

Three typical examples of residual impression at peak load of 5 mN, 10 mN, and 15 mN are shown in Fig. 10. It can be seen from Fig. 10(a) that although the residual impression seems to be very irregular, there is no obvious extrusion and crack around the indentation. Hence, the HPPT and dislocation burst are the possible cause of the initial pop-in event in the *L-ε* curve. Dislocation formation in silicon has so far been shown only a small effect on the deformation compared to other materials especially when a small indenter is used. The present MD simulations indicate that the dislocation burst occurs behind of occurrence of HPPT, and the dislocations slightly contribute to the plastic deformation. Defect propagation and HPPT are reported to be mutually exclusive, competing deformation mechanism at low peak load^24^, however this fact may be due to the large radius of indenter used. Thus, it is rational that the HPPT is responsible for the observed initial pop-in event synthetically considering the experiments and MD simulation.

In Fig. 10(b), regular residual impression appears at peak load of 10 mN. The distinct extrusion around the indentation is observed, and there is no obvious cracking on the surface. Increasing peak load to 10 mN, three pop-in events are observed. Therefore, it is reasoned that the second and third pop-in events are linked to the sudden extrusion of ductile material. At peak load to 15 mN, three major cracks appear on the surface accompanying with the fourth pop-in event, as shown in Fig. 10(c). So, the surface crack is responsible for the fourth pop-in event in the *L-ε* curve. Another possible cause of the pop-in is the subsurface crack, which is commonly found in experiments and the present MD simulations. Until now, there is no report on the relationship between the crack in subsurface and the pop-in. Present MD simulations also indicate that the median crack at the bottom of the transformation region produces no detectable pop-in in *L-ε* curve. Further *in situ* nanoindentation and XTEM characteristic are needed to support or refute these possibilities, and validate the present MD simulations.

## Discussion

### Mechanism of plastic deformation

The versatile characteristics of silicon lead to the plastic deformation mechanism under contact load remaining unclear. The present MD simulations reveal that single crystal silicon can deform not only in a ductile manner, driven by HPPT and dislocations, but also in a brittle manner, driven by direct cracking at the nanoscale. The key factor governing deformation mode is the indentation strain. Dislocation- and cracking-mediated deformation are detected at large indentation strain. As a matter of fact, very few dislocations are observed, and only one median crack is observed in the subsurface, which suggests that the contribution of dislocations and cracks to the plastic deformation is almost negligible. Therefore, phase transformation is the dominant deformation mechanism and is the single mode of mechanical deformation when the indentation strain is less than 0.107 in simulation, suggesting a defect-free region at small *ε* during nanoindentation. This single-mode mechanical deformation at small load has been proven by a recent experiment^2^. A defect free region is necessary for engineering applications, for example, defect free CMP (the chemical mechanical polishing) and the ultraprecision grinding of silicon have been developed based on this principle^34^.

At large indentation strains, a large number of dislocations, major slips, cracking and extensive crystalline damage are generally found beneath the indenter in experiments^1, 2, 16, 24^. Although the occurrences of dislocations and cracks predicted by present MD simulations are less than that observed in experiments, the morphology of the deformation region in our simulations is consistent with experiments, *i.e*. a phase transformation region appears near the surface and forms an impression, and the dislocations and cracks develop beneath the transformation region accompanied by an extrusion on the surface. These results are illustrated by the cross-sectional views of deformation region in MD simulations (Figs 4 and 6) and the XTEM images in experiments^1, 2, 16, 24^.

The present MD simulation results demonstrate that the piecewise plastic deformation of silicon is related to the indentation strain. When the indentation strain ranges from 0.067 to 0.115, the pristine diamond-structured silicon continuously transforms into crystalline Si-II and bct5 phases, which results in the rapid expansion of the Si-II and bct5 phase volume. Once the indentation strain increases to over 0.115, the crystalline-to-amorphous transformation and the extrusion of α-Si continuously proceed. In this case, the flow and extrusion of α-Si are linked to the plastic deformation. The dislocations nucleate (at *ε* = 0.107) after the occurrence of the phase transformation (at *ε* = 0.067) and shortly before the appearance of α-Si (*ε* = 0.115). A large shear stress is generally known to drive the nucleation of dislocations and amorphization^45, 53^. Therefore, the dislocations and amorphous phase appear almost simultaneously.

The deformation behavior can be closely related to the stress state. At small *ε*, the large hydrostatic pressure and associated small shear stress drives the Si-I to Si-II and Si-I to bct5 phase transformations. As the penetration proceeds, the continuously increasing shear stress and hydrostatic pressure facilitate the nucleation of dislocations and amorphization, and following extrusion of α-Si and crack.

### Pop-ins

The occurrence of pop-in events during loading in the nanoindentation of silicon is a very interesting and controversial topic. The mechanism behind the pop-ins is far less clear due to the lack of accurate *in situ* structures and defect detection during nanoindentation. It is known from previous studies that three events are responsible for pop-ins. (i) The Si-I to Si-II transformation is linked to the occurrence of pop-ins. Convincing evidence comes from the pop-in observed in DAC experiments via *in situ* electrical characterization. (ii) Pop-in can be caused by sudden extrusion, rather than the formation of the Si-II phase, which is based on the observation that the phase transformation is detected prior to the occurrence of the pop-in event^1, 15, 58, 59^. (iii) Dislocation burst or cracking induce the pop-in event^9, 10, 24^.

In the present paper, the occurrence of multiple pop-in events in *L-ε* curve was reported, and three mechanisms were identified to be responsible for the observed pop-in events based on the combined study using MD simulation and experiment. In the first mechanism, the Si-I to Si-II and Si-I to bct5 phase transformations induce the initial pop-in, which define the incipient plasticity. This result supports the above-mentioned claim (i). The formation and extrusion of α-Si outside the indentation cavity are responsible for the pop-ins at high load (Pop-in B in MD simulation, and second and third pop-in in experiments), as the second mechanism. This is similar to the abovementioned claim (ii). The differences result from the different extrusion materials. Present MD simulation indicate that the extrusion of α-Si, rather than Si-II, occurs outside the indentation cavity. The extrusion of Si-II is based on the assumption that the Si-II phase is the single high-pressure phase in the transformation region, in which convincing evidence is lacked. In fact, the observed bct5 phase and α-Si have higher probability for extrusion because these two phases distribute directly alongside the indenter. Present MD simulations show that dislocation burst and median crack at the bottom of the transformation region are not linked to pop-in events because few dislocations and only one median crack is found beneath the transformation region. As the nanoindentation strain continues to increase, the major cracks on the surface induce the pop-in at extreme high load (fourth pop-in in present experiment), as the third pop-in mechanism. This mechanism is found in present experimental nanoindentation. Although first two mechanisms and the median crack are found in the present MD simulation, the third pop-in mechanism, surface cracking, still cannot be predicted. The improved MD simulations and more experiments are need to further our understanding of this mechanism.

At experimental conditions, not all the pop-ins can be detected, because of the influence of the indenter geometry and size, the loading rate, the peak load, and the limited instrumental sensitivity, which may result in different interpretations for pop-ins. For example, the Pop-in B predicted by the present MD simulations is difficult to detect during nanoindentation using a spherical indenter with large radius, because of the so large critical indentation strain. In contrast, the critical strain can be easily achieved by a sharp indenter, such as Vickers, Berkovich and cube corner indenters. In fact, the kink pop-in and direct amorphization with Berkovich indenter have been reported, which indirectly verifies the present results. The recent nanoindentation experiments using the small indenter and ultra-low loads confirm the HPPT is responsible for the first pop-in ref. 56, which is consistent with the present MD simulations. In our experimental nanoindentation, the small indenter, slow loading rate and large peak load promote the occurrence of multiple pop-in events especially the pop-in events at low load.

## Conclusion

In the present paper, by combined study using MD simulation and experiment, the occurrence of multiple pop-in events in *L-ε* curve was found during nanoindentation of single crystal silicon with a spherical indenter. Three distinct mechanisms are identified to be responsible for the pop-in events. In the first mechanism, the Si-I to Si-II and Si-I to bct5 transformations induce the first pop-in, which defines the incipient plasticity. The formation and extrusion of α-Si outside the indentation cavity are responsible for the subsequent pop-in event at high load, as the second mechanism. The major cracks on the surface induces the pop-in event at extreme high load, as the third pop-in mechanism. Although dislocation burst and median crack are also observed at large indentation strain, these events produce no detectable pop-in event in *L-ε* curve according to the MD simulations.

We also show that single crystal silicon can deform not only in a ductile manner, driven by HPPT and dislocations, but also in a brittle manner, driven by direct cracking. The key factor governing the deformation mode is the indentation strain. Even so, very few dislocations are observed, and only one median crack is observed at the bottom of the transformation region, suggesting that the contributions of dislocations and cracks to the plastic deformation are almost negligible. We emphasize that phase transformation is the dominant deformation mode and is the single mode of mechanical deformation with small indentation strain during nanoindentation, suggesting the presence of a defect-free region. This work advances our understanding of the nature of single crystal silicon with respect to the elastic-plastic transition, pop-ins, phase transformations and amorphization, which are of great significance for silicon science and technologies.

## Electronic supplementary material

supplemental material

## References

[1] Bradby JE, Williams JS, Wong-Leung J, Swain MV, Munroe P (2000). Transmission electron microscopy observation of deformation microstructure under spherical indentation in silicon. Appl. Phys. Lett..

[2] Wong S, Haberl B, Williams JS, Bradby JE (2015). Phase transformation as the single-mode mechanical deformation of silicon. Appl. Phys. Lett..

[3] Zarudi I, Zou J, Zhang LC (2003). Microstructures of phases in indented silicon: A high resolution characterization. Appl. Phys. Lett..

[4] Gerbig YB, Stranick SJ, Cook RF (2011). Direct observation of phase transformation anisotropy in indented silicon studied by confocal Raman spectroscopy. Phys. Rev. B.

[5] Bradby JE, Williams JS, Wong-Leung J, Swain MV, Munroe P (2001). Mechanical deformation in silicon by micro-indentation. J. Mater. Res.

[6] Zhang LC, Zarudi I (2001). Towards a deeper understanding of plastic deformation in mono-crystalline silicon. Int. J. Mech. Sci..

[7] Kiran MSRN (2015). Temperature-dependent mechanical deformation of silicon at the nanoscale: Phase transformation versus defect propagation. J. Appl. Phys..

[8] Mylvaganam K, Zhang LC (2011). Nanotwinning in monocrystalline silicon upon nanoscratching. Scripta Mater..

[9] Minor AM (2005). Room temperature dislocation plasticity in silicon. Philos. Mag..

[10] Chrobak D (2011). Deconfinement leads to changes in the nanoscale plasticity of silicon. Nat. Nanotechnol..

[11] Suzuki T, Ohmura T (1996). Ultra-microindentation of silicon at elevated temperatures. Philosophical Magazine A.

[12] Gilman JJ (1993). Why silicon is hard. Science.

[13] Gridneva IV, Milman YV, Trefilov VI (1972). Phase transition in diamond-structure crystals during hardness measurements. Physica status solidi (a).

[14] Clarke DR (1988). Amorphization and conductivity of silicon and germanium induced by indentation. Phys. Rev. Lett..

[15] Bradby JE, Williams JS, Swain MV (2003). *In situ* electrical characterization of phase transformations in Si during indentation. Phys. Rev. B.

[16] Ruffell S, Bradby JE, Williams JS, Munroe P (2007). Formation and growth of nanoindentation-induced high pressure phases in crystalline and amorphous silicon. J. Appl. Phys..

[17] Jang JI, Lance MJ, Wen SQ, Tsui TY, Pharr GM (2005). Indentation-induced phase transformations in silicon: influences of load, rate and indenter angle on the transformation behavior. Acta Mater..

[18] Juliano T, Domnich V, Gogotsi Y (2004). Examining pressure-induced phase transformations in silicon by spherical indentation and Raman spectroscopy: A statistical study. J. Mater. Res..

[19] Chang L, Zhang LC (2009). Deformation mechanisms at pop-out in monocrystalline silicon under nanoindentation. Acta Mater..

[20] Das CR (2010). Direct observation of amophization in load rate dependent nanoindentation studies of crystalline Si. Appl. Phys. Lett..

[21] Zarudi I, Zhang LC, Cheong W, Yu TX (2005). The difference of phase distributions in silicon after indentation with Berkovich and spherical indenters. Acta Mater..

[22] Gerbig YB, Stranick SJ, Morris DJ, Vaudin MD, Cook RF (2009). Effect of crystallographic orientation on phase transformations during indentation of silicon. J. Mater. Res..

[23] Gerbig Y.B., Stranick S.J. & Cook R.F. Measurement of residual stress field anisotropy at indentations in silicon. *Scripta Mater*. **63**, 512–515 (2010).

[24] Wong S, Haberl B, Williams JS, Bradby JE (2015). The influence of hold time on the onset of plastic deformation in silicon. J. Appl. Phys..

[25] Zarudi I, Zhang LC (1999). Structure changes in mono-crystalline silicon subjected to indentation-experimental findings. Tribol. Int..

[26] Ruffell S, Haberl B, Koenig S, Bradby JE, Williams JS (2009). Annealing of nanoindentation-induced high pressure crystalline phases created in crystalline and amorphous silicon. J. Appl. Phys..

[27] Zhang N (2011). Deformation mechanisms in silicon nanoparticles. J. Appl. Phys..

[28] Ostlund F (2009). Brittle-to-Ductile Transition in Uniaxial Compression of Silicon Pillars at Room Temperature. Adv. Funct. Mater..

[29] Stauffer DD (2012). Strain-hardening in submicron silicon pillars and spheres. Acta Mater..

[30] Zarudi. I, Zhang LC (1998). Effect of ultraprecision grinding on the microstructural change in silicon monocrystals. J. Mater. Proc. Tech..

[31] Wang Y (2007). Formation mechanism of nanocrystalline high-pressure phases in silicon during nanogrinding. Nanotechnology..

[32] Wu YQ, Huang H, Zou J, Zhang LC, Dell JM (2010). Nanoscratch-induced phase transformation of monocrystalline Si. Scripta Mater..

[33] Zhang ZY, Wu YQ, Guo DM, Huang H (2011). Phase transformation of single crystal silicon induced by grinding with ultrafine diamond grits. Scripta Mater..

[34] Zhang Z (2016). Nanoscale solely amorphous layer in silicon wafers induced by a newly developed diamond wheel. Sci. Rep.-Uk.

[35] Cleri F, Ishida T, Collard D, Fujita H (2010). Atomistic simulation of plasticity in silicon nanowires. Appl. Phys. Lett..

[36] Sun J, Ma A, Jiang J, Han J, Han Y (2016). Orientation-dependent mechanical behavior and phase transformation of mono-crystalline silicon. J. Appl. Phys..

[37] Goel S, Faisal NH, Luo X, Yan J, Agrawal A (2014). Nanoindentation of polysilicon and single crystal silicon: Molecular dynamics simulation and experimental validation. Journal of Physics D: Applied Physics.

[38] Du X (2015). Molecular dynamics investigations of mechanical behaviours in monocrystalline silicon due to nanoindentation at cryogenic temperatures and room temperature. Sci. Rep.-Uk.

[39] Cheong W, Zhang LC (2000). Molecular dynamics simulation of phase transformations in silicon monocrystals due to nano-indentation. Nanotechnology.

[40] Lin YH, Jian SR, Lai YS, Yang PF (2008). Molecular dynamics simulation of nanoindentation-induced mechanical deformation and phase transformation in monocrystalline silicon. Nanoscale Res. Lett..

[41] Kim DE, Oh SI (2008). Deformation pathway to high-pressure phases of silicon during nanoindentation. J. Appl. Phys..

[42] Kim DE, Oh SI (2006). Atomistic simulation of structural phase transformations in monocrystalline silicon induced by nanoindentation. Nanotechnology.

[43] Ivashchenko VI, Turchi PEA, Shevchenko VI (2008). Simulations of indentation-induced phase transformations in crystalline and amorphous silicon. Phys. Rev. B.

[44] Eyben P (2010). Analysis and modeling of the high vacuum scanning spreading resistance microscopy nanocontact on silicon. J. Vac. Sci. Technol. B.

[45] Sun J (2014). Phase transformations of mono-crystal silicon induced by two-body and three-body abrasion in nanoscale. Comp. Mater. Sci..

[46] Sanz-Navarro CF, Kenny SD, Smith R (2004). Atomistic simulations of structural transformations of silicon surfaces under nanoindentation. Nanotechnology.

[47] Gerbig YB, Michaels CA, Forster AM, Cook RF (2012). *In situ* observation of the indentation-induced phase transformation of silicon thin films. Phys. Rev. B.

[48] Albe K, Erhart P (2005). Analytical potential for atomistic simulations of silicon, carbon, and silicon carbide. Phys. Rev. B.

[49] Pastewka L, Klemenz A, Gumbsch P, Moseler M (2013). Screened empirical bond-order potentials for Si-C. Phys. Rev. B.

[50] Goel S, Kovalchenko A, Stukowski A, Cross G (2016). Influence of microstructure on the cutting behaviour of silicon. Acta Mater..

[51] Schneider T, Stoll E (1978). Molecular-dynamics study of a three-dimensional one-component model for distortive phase transitions. Phys. Rev. B.

[52] Plimpton S (1995). Fast parallel algorithms for short-range molecular-dynamics. J. Comput. Phys..

[53] Han J, Xu S, Sun J, Fang L, Zhu H (2017). Pressure-induced amorphization in the nanoindentation of single crystalline silicon. Rsc Adv..

[54] Xiao C (2017). Effect of crystal plane orientation on tribochemical removal of monocrystalline silicon. Sci. Rep.-Uk.

[55] Takahagi. T, Nagai I, Ishitani A, Kuroda H, Nagasawa Y (1988). The formation of hydrogen passivated silicon single-crystal surfaces using ultraviolet cleaning and HF etching. J. Appl. Phys..

[56] Chang L, Zhang LC (2009). Mechanical behaviour characterization of silicon and effect of loading rate on pop-in: A nanoindentation study under ultra-low loads. Mat. Sci. Eng. A-Struct..

[57] Hebbache M, Zemzemi M (2003). Nanoindentation of silicon and structural transformation: Three-dimensional contact theory. Phys. Rev. B.

[58] Abram R, Chrobak D, Nowak R (2017). Origin of a Nanoindentation Pop-in Event in Silicon Crystal. Phys. Rev. Lett..

[59] Haq AJ, Munroe PR (2009). Phase transformations in (111) Si after spherical indentation. J. Mater. Res..

[60] Li J, Van Vliet KJ, Zhu T, Yip S, Suresh S (2002). Atomistic mechanisms governing elastic limit and incipient plasticity in crystals. Nature.

[61] Chrobak D, Kim KH, Kurzydłowski KJ, Nowak R (2013). Nanoindentation experiments with different loading rate distinguish the mechanism of incipient plasticity. App. Phys. Lett..

