# Supplementary material for: Nanoindentation Induced Deformation and Pop-in Events in a Silicon Crystal: Molecular Dynamics Simulation and Experiment
Source: Sci Rep. 2017 Aug 31;7:10282. doi: 10.1038/s41598-017-11130-2 (PMC5578969; doi:10.1038/s41598-017-11130-2)
Supplement: Supplementary file 1 — supplemental material [file 41598_2017_11130_MOESM1_ESM.pdf]

**Supplemental Material to the Letter**

# Nanoindentation Induced Deformation and Pop-in Events in a Silicon Crystal: Molecular Dynamics Simulation and Experiment

**Sun Jiapeng[1]*, Li Cheng[1], Jing Han[2]*, Aibin Ma[1], Liang Fang[3]**

1 College of Mechanics and Materials, Hohai University, Nanjing 210098, Jiangsu Province, PR China

2 School of Mechanical and Electrical Engineering, China University of Mining and Technology, Xuzhou 221116, Jiangsu Province, PR China

3 State Key Laboratory for Mechanical Behavior of Materials, Xi'an Jiaotong University, Xi'an 710049, Shaanxi Province, PR China

E-mail: sun. jiap@gmail.com; hanjing@cumt.edu.cn

## Method of identifying the structural phases

During nanoindentation, five phases (Si-I, Si-II, Si-III, Si-XII and bct5 phase) were observed and classified in the previous experiment [1] and simulation results [2-4]. The structure characteristics of five crystal phases is summarized in Table S1 along with the typical transition pressure. To identify these phases, the techniques of modified coordination number (CN), radial distribution function (RDF) and bond angle distribution function (ADF) are applied jointly in this work. Although RDF and ADF are widely employed to classify the crystal structures, both the techniques become powerless when different phases are mixed together in a small volume as in the nanoindentation. In this work, CN is firstly applied to identify the phase structures and profile the phase region and phase distribution, because CN just consider the surrounding environments of an atom with a radius of 0.35nm in current work. The CN also provides a visualization method of structural phases. RDF and ADF are further used to verify and characterize the detailed structures of the high-pressure phase.

Si-I phase has four nearest neighbors at a distance of 0.235nm at ambient pressure. Si-II has four nearest neighbors at a distance of 0.242nm and two at only slightly larger distance of 0.257nm. bct5 phase has one neighbor at a distance of 0.231nm and four at 0.244nm. Hence, Si-I, Si-II and bct5 phase can be distinguished

easily just considering the nearest neighbors with the maximum bond length of 0.28nm in the present paper. Si-III phase and Si-XII both have four nearest neighbors within the distance of 0.237nm and 0.239nm respectively. Hence, Si-III/XII cannot be distinguished from Si-I just considering the nearest neighbors. However, Si-III has a unique non-bonded fifth neighbor at 0.341nm at 2GPa, and Si-XII has a unique one at 0.323nm or 0.336nm at 2GPa [1], while Si-I has twelve non-bonded second neighbors at 0.383nm. Based on this difference, fourfold coordinated silicon atoms, which have unique one non-bonded neighbor in the range from 0.28nm to 0.35nm, are assumed as metastable Si-III/XII phase with fourfold coordination. This phase identified method has been successfully used to distinguish the phase structures during nanoindentation [2-4].

**Table S1** High pressure phases of silicon during nanoindentation in Refs. [5-9]

| Phase | Lattice structure | Lattice parameter (Å) | *c/a* | Relative volume | Transition pressure |
|---|---|---|---|---|---|
| Si-I | Diamond cubic (dc) | 5.429 | 1 | 1 | 1-12.5 GPa |
| Si-II | Body-centered-tetragonal (β-Sn) | a=4.686 c=2.587 | 0.55 | 0.71 | 9-16 Gpa |
| Si-III | Body-centered cubic (bc8) | a=6.64 | 1 | 0.92 | Unloading to 2.1-0 GPa |
| Si-XII | Rhombohedral (r8) | a=5.609 γ=110.07 | | 0.9 | Unloading to 12-2 GPa |
| bct5 | Body-centered-tetragonal | a=3.313 c=5.959 | 1.80 | 0.85 | 12.6 GPa |

When the phases have been classified by CN, RDF and ADF are further used to verify and characterize the detailed structures of the high-pressure phase within the congregating single-phase region identified by CN. Because the typical dimension of high pressure phase is ~1nm, the atoms located in a skin of 0.5nm surrounding these single-phase region are considered into the computation of both RDF and ADF, but taken into account the neighbors atoms. This computation region for RDF and ADF is

sufficiently large to characterize the phase structure within a cut-off distance of 0.5nm.

**Structural Characterization**

The RDF and ADF of atoms located at selected Si-II and bct5 cuboid region are computed to verify the high-pressure phase identified by the CN at $\varepsilon$= 0.109 as shown in Fig. S1. For comparison, the RDF peaks of bct5 phase calculated by plane wave pseudopotential method [10] and Si-II phase calculated by Tersoff potential [11] are also shown in Figure. Although the RDF peaks have slightly shifted which is attributed to the different calculated method and the severe deformation of the high-pressure phases. This result is agreed with that predicted by the MD simulation of nanoindentation using Tersoff potential [12] and vindicates our MD simulation using screened empirical bond-order potentials. The RDF and ADF peaks confirm the formation of Si-II and bct5 phase. And the broadened and shifted RDF and ADF peaks indicate that the Si-II and bct5 phases undergo severe deformation. Fig.S2 shows the network structure of the Si-II and bct5 phases. The fourfold ring and sixfold ring confirm the Si-II and bct5 phases respectively.

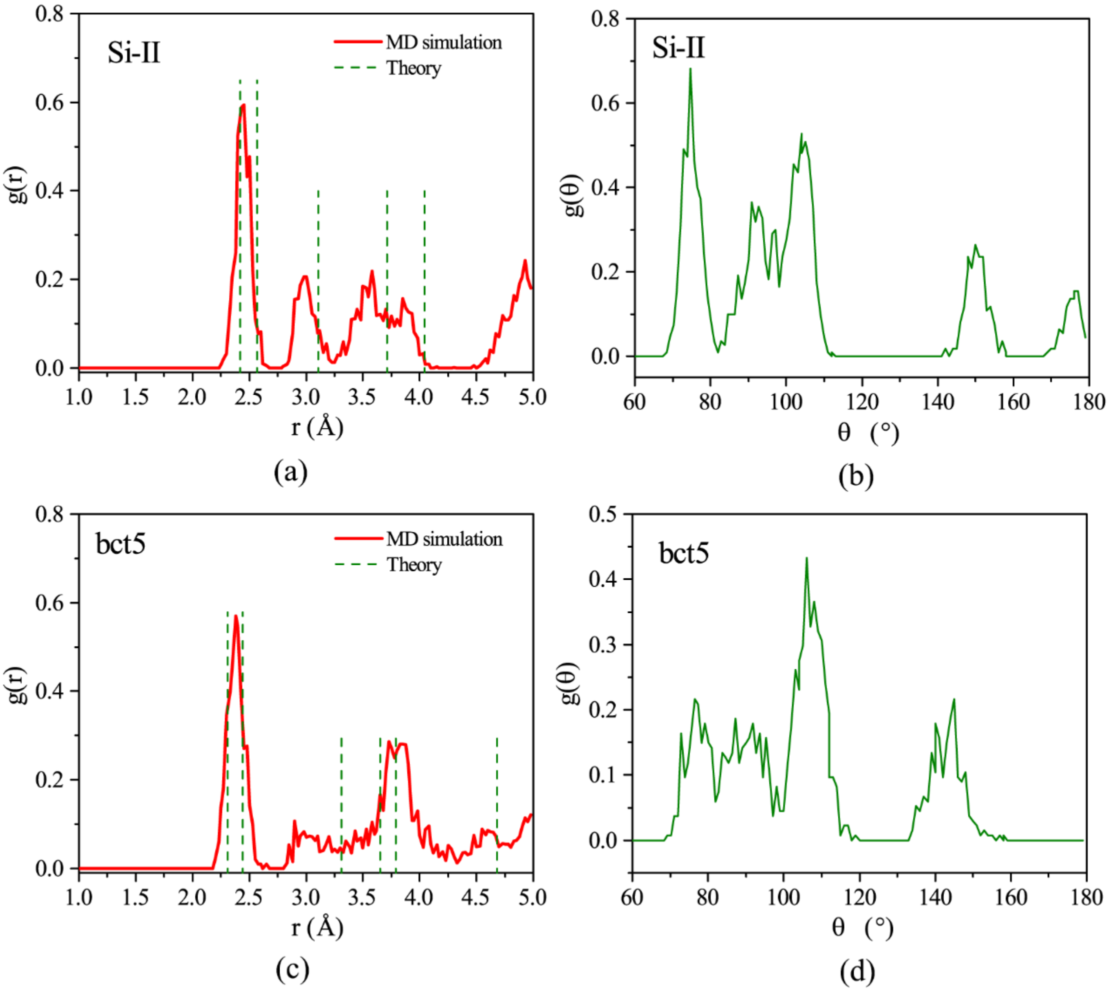


Fig. S1 (a) RDF of the Si-II, (b) ADF of the Si-II, (c) RDF of the bct5, (d) ADF of the bct5 at $\varepsilon$= 0.109.

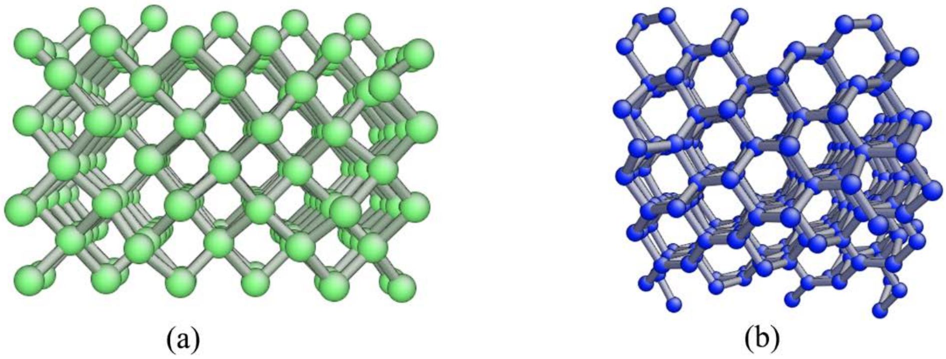


Fig. S2 The structures of (a) the Si-II and (b) the bct5, (d) ADF of the bct5 at $\varepsilon$= 0.109.

Fig.S3 shows the RDF and ADF of the a-Si phase in a selected cuboid region at $\varepsilon$= 0.2. The disappeared third RDF peak, broadened RDF and ADF peaks confirm the amorphous characteristic. The first RDF peak appears at the distance of 0.24nm which

is slightly larger than that of the perfect cubic diamond structure silicon (0.235nm). The centered peak of ADF is separated into two peak at 76.50° and 103° respectively, and there is a small peak near 58.50°. This is very different from the perfect cubic diamond structure silicon, of which the ADF has single peak at 109°.The careful calculation shows the average coordination number of the a-Si phase is 5.63, the proportion of 5- and 6-coordinate silicon atoms approaches ~79.50%, and the volume of per atom of a-Si is 0.66 relative to the zero-temperature diamond structure. Fig. S4 shows the network structure of the a-Si.

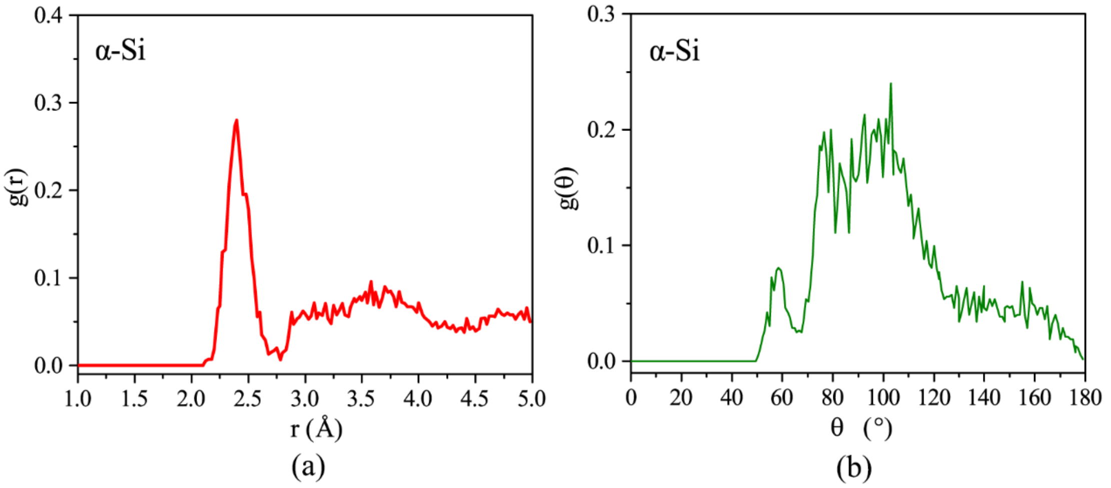


Fig. S3 (a) RDF and (b) bct5 of the a-Si at $\varepsilon$= 0.2.

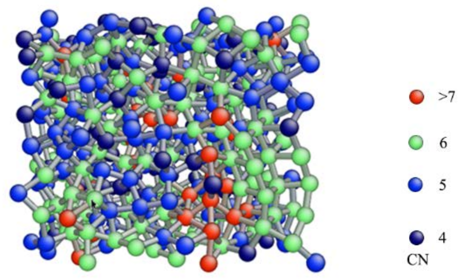


Fig. S4 The structures of the a-Si at $\varepsilon$= 0.2.

# References:

[1] Piltz R.O., Maclean J.R., Clark S.J., Ackland G.J., Hatton P.D., Crain J. Structure and properties of silicon-xii - a complex tetrahedrally bonded phase. Phys Rev B. 1995;52:4072-85.

[2] Mylvaganam K., Zhang L.C., Eyben P., Mody J., Vandervorst W. Evolution of metastable phases in silicon during nanoindentation: Mechanism analysis and experimental verification. Nanotechnology. 2009;20.

[3] Kim D.E., Oh S.I. Atomistic simulation of structural phase transformations in monocrystalline silicon induced by nanoindentation. Nanotechnology. 2006;17:2259-65.

[4] Abram R., Chrobak D., Nowak R. Origin of a nanoindentation pop-in event in silicon crystal. Phys Rev Lett. 2017;118:95502.

[5] Goel S., Luo X., Agrawal A., Reuben R.L. Diamond machining of silicon: A review of advances

in molecular dynamics simulation. International Journal of Machine Tools and Manufacture. 2015;88:131-64.
[6] Ivashchenko V.I., Turchi P.E.A., Shevchenko V.I. Simulations of indentation-induced phase transformations in crystalline and amorphous silicon. Phys Rev B. 2008;78:35205.
[7] Ge D.B., Domnich V., Gogotsi Y. High-resolution transmission electron microscopy study of metastable silicon phases produced by nanoindentation. J Appl Phys. 2003;93:2418-23.
[8] Jamieson J.C. Crystal structures at high pressures of metallic modifications of silicon and germanium. Science. 1963;139:762.
[9] Wong S., Haberl B., Williams J.S., Bradby J.E. Phase Transformation Dependence on Initial Plastic Deformation Mode in Si via Nanoindentation. Exp Mech. 20161-7.
[10] Boyer L.L., Kaxiras E., Feldman J.L., Broughton J.Q., Mehl M.J. New low-energy crystal-structure for silicon. Phys Rev Lett. 1991;67:715-8.
[11] Halicioglu T., Tiller W.A., Balamane H. Comparative study of silicon empirical interatomic potentials. Phys Rev B. 1992;46:2250-79.
[12] Sun J., Fang L., Han J., Han Y., Chen H., Sun K. Phase transformations of mono-crystal silicon induced by two-body and three-body abrasion in nanoscale. Comp Mater Sci. 2014;82:140-50.
